# Feminization of the medical workforce in low-income settings; findings from surveys in three African capital cities

**DOI:** 10.1186/s12960-015-0064-9

**Published:** 2015-07-31

**Authors:** Giuliano Russo, Luzia Gonçalves, Isabel Craveiro, Gilles Dussault

**Affiliations:** International Health and Biostatistics Unit, Instituto de Higiene e Medicina Tropical, Universidade Nova de Lisboa, Lisbon, Portugal; WHO Collaborating Centre for Health Workforce Policies and Planning, Lisbon, Portugal; Global Health and Tropical Medicine Global Health and Tropical Medicine, GHTM, Lisbon, Portugal; Centro de Estatística e Aplicações da Universidade de Lisboa, Lisbon, Portugal

## Abstract

**Background:**

Women represent an increasingly growing share of the medical workforce in high-income countries, with abundant research focusing on reasons and implications of the phenomenon. Little evidence is available from low- and middle-income countries, which is odd given the possible repercussion this may have for the local supply of medical services and, ultimately, for attaining universal health coverage.

**Methods:**

Drawing from secondary analysis of primary survey data, this paper analyses the proportion and characteristics of female physicians in Bissau, Maputo and Praia, with the objective of gaining insights on the extent and features of the feminization of the medical workforce in low- and middle-income settings. We used descriptive statistics, parametric and non-parametric test to compare groups and explore associations between different variables. Zero-inflated and generalized linear models were employed to analyse the number of hours worked in the private and public sector by male and female physicians.

**Results and discussion:**

We show that although female physicians do not represent yet the majority of the medical workforce, feminization of the profession is under way in the three locations analysed, as women are presently over-represented in younger age groups. Female doctors distribute unevenly across medical specialties in the three cities and are absent from traditionally male-dominated ones such as surgery, orthopaedics and stomatology. Our data also show that they engage as much as their male peers in private practice, although overall they dedicate fewer hours to the profession, particularly in the public sector.

**Conclusions:**

While more research is needed to understand how this phenomenon affects rural areas in a broader range of locations, our work shows the value of exploring the differences between female and male physicians’ engagement with the profession in order to anticipate the impact of such feminization on national health systems and workforces in low- and middle-income countries.

**Electronic supplementary material:**

The online version of this article (doi:10.1186/s12960-015-0064-9) contains supplementary material, which is available to authorized users.

## Introduction

The increasing proportion of female physicians and its consequences for healthcare delivery systems – often referred to as “feminization of the medical workforce” – have been extensively explored in the human resources for health literature [1]. Some authors have argued that the roots of the phenomenon are in the changing features of the profession that make it no longer as attractive to men [2,3] or in female students’ greater (easier) access to medical training [3]. In high-income countries, the number of women entering medical education is superior to that of men. Newly graduated male physicians represent the majority of new entrants in the medical workforce in the United Kingdom [4], Canada [5], France [6] and Japan [7]. The proportion of female physicians is also rising in countries like Israel [8]. Consolidated international data are infrequently updated but nonetheless point towards substantial differences in the gender distribution of the physicians’ workforce worldwide (see Additional file 1: Table S1 in the statistical annex).

Levinson and Lurie suggest that feminization will bring changes in four dimensions of the medical profession: in the patient–physician relationship, in the local delivery of care, in the global delivery of care to the society as a whole and in the medical profession itself [9]. Empirical evidence from high-income countries shows that female physicians work fewer hours, particularly at a younger age, see less patients than their male peers [10], are less inclined to work in rural areas [11] and concentrate disproportionally in “soft” specialties [7,8]. This has triggered concerns on the potential consequences for the availability and accessibility of medical services to the population [4]. Evidence also exists of positive aspects of female physicians’ different practice patterns, such as spending more time with their patients [12], writing fewer prescriptions and referring cases more often [1].

The reasons for female physicians’ smaller workload output have not been fully understood yet. Parenthood appears to play a crucial role in the number of hours female professionals dedicate to the profession [13]; they seem to be paid less than their male counterparts [14] and appear to be spending more time with individual patients [12]. In Norway, it was observed that having children reduces working hours by 80% among female physicians but has no effect on male peers’ time [15]. Some authors have put forward the hypothesis that, while female physicians have for quite some time already given preference to a lighter workload, only recently male ones started seeking a less demanding work–life balance, and this would explain the current differences between sexes [16].

However, a dearth of studies exists on the phenomenon and its consequences in resource-poor settings. Feminization of the medical workforce is an established phenomenon in South Africa [2]. A 2013 study found that female candidates represent the majority of medical school graduates in Goa, India, but that only 41% of them were actually practising the profession [17]. The proportion of female physicians in a group of African, Asian and Latin American low- and middle-income countries is low because of their more limited access to training funds and to other non-financial benefits [18]. Female physicians’ engagement with private sector activities, a phenomenon of increasing importance in low-income countries [19], is also understudied.

The present paper analyses the proportion and characteristics of female physicians in three capital cities in Lusophone Africa with the objective of gaining insights on extent, features and implications of the medical workforce’s feminization in the light of the universal health coverage (UHC) objectives. If women in low-income countries adopt the same behaviours of their peers in high-income countries, current deficits of medical professionals will likely increase, with obvious implication for the attainment of the UHC goals.

The three capital cities were originally selected for their common history and similarities in their legal systems and organization of public sector and national healthcare system, as well as for being at different stages of development, which is believed to offer an insight on the evolution of feminization of the workforce [20]. Bissau, Guinea-Bissau’s capital, had a proportion of 3.27 physicians per 10 000 population in 2012, with low GDP per capita and an underdeveloped formal private health sector, though private services were also offered – often illegally – within public facilities [21] (Table 1). Guinea-Bissau’s health system is, to the current days, severely underfunded and donor dependent, with health expenditures per capita of USD 38 in 2010, and a large primary care network relying on seven secondary-care hospitals where most of the physicians operate.

**Table 1:** **Selected characteristics for the three study locations**

**Characteristics**	**Bissau (Guinea-Bissau)**	**Maputo (Mozambique)**	**Praia (Cape Verde)**
Country’s GDP per capita (PPP)^a^	1186	942	3984
Total health expenditures per capita (current prices, 2010)	46.9	21.3	154.6
Position in the HDI (out of 187)^b^	176th	184th	133rd
Physicians registered in the country^c^	172	1105	400
Physicians residing in the countries’ capital cities^c^	127	487	131
Population in capital cities^c^	387 908	1 178 116	131 453
Physician density in capital cities (per 100 000 population)	3.27	6.64	9.96

*Sources*: ^a^ The World Bank (2012); ^b^ UNDP (2011); ^c^ National Medical Councils (2012), National Statistical Institutes.

Maputo, capital of Mozambique, is the largest city of the sample and had 6.64 physicians per 10 000 population at the time of the study; demand for both public and private services is sustained, and the private sector is rapidly developing, following the country’s natural-resources-related economic growth. Maputo Central Hospital is Mozambique’s largest treatment institution for a network of 3 central hospitals, 7 provincial ones and 45 rural hospitals based in provincial capital cities. Healthcare standards and cost of living in Maputo are increasingly distancing themselves from the rest of the country [22]. In Cape Verde’s capital city, Praia, there were 9.96 physicians per 10 000 inhabitants in 2012; public sector salaries are comparatively high, representing almost five times the national gross domestic product per capita. The only one of the three capitals to have avoided post-independence civil war, Cape Verde is one of Africa’s few middle-income countries [23]. Its health system is comparatively the most developed of the three countries, with two central hospitals in the main islands of Santiago and São Vicente and three regional hospitals in the rest of the archipelago.

## Methods

### Data collection

This paper draws on a secondary analysis of primary survey data collected for a study in the three cities on physicians’ simultaneous engagement in multiple professional activities in the public as well as private sector (dual practice); the data analysed here are the same, and a more detailed description of the sample selection strategy and the methods used to build that data set are available in a separate article [20].

Surveys were used to collect data using a sampling frame from all physicians in Bissau and Praia, and from a random sample in Maputo, containing 31 questions on their demographic profile, allocation of time across professional activities, public and private sector work characteristics, and regulation of the profession (see the survey questionnaire in Additional file 2). The surveys were conducted by three separate teams of 10 local data collectors with a health sector background, trained and supported by a team of researchers from the Institute of Hygiene and Tropical Medicine (IHMT). The survey questionnaire was administered in person; in Bissau and Praia, response rates were around 80%, for a final number of respondents of 96 and 110, respectively. In Maputo, where the physician population was considerably larger, we surveyed a simple random sample of 125 physicians drawn from the Ministry of Health and Medical Council databases. Overall, in the three cities, 51.8% of those in the lists accepted to participate, 7.1% refused and 41.1% could not be located as they were either posted somewhere else, dead or away at the time of data collection. No systematic difference was identified between respondents and non-respondents.

## Data analysis

Our data set included information on age, sex, marital status, number of household dependents, length of time since graduating, whether working outside the city in which the physician lives, specialty, place of work and engagement with dual practice. The questions for public sector physicians included the level (primary, secondary, tertiary) of the institution, net monthly salary and whether they gained extra income above their basic salary. For private sector physicians, questions included place of work and reasons for not working more hours in the private sector. All information was disaggregated by gender and the main variables used in the statistical analysis are presented in Additional file 1: Table S2 (statistical annex).

In addition to descriptive statistics, parametric tests and non-parametric test (Mann–Whitney–Wilcoxon and Kruskal–Wallis tests, respectively) were used to compare groups and explore associations between different variables (chi-square test or the alternative Fisher exact test). Confidence intervals for proportions (for example, physicians in dual practice) were obtained using Wilson and Agresti–Coull methods [24]. SPSS and EpiTools [25] were used to explore our data set. Zero-inflated (ZI) models were explored taking into account the nature of the distributions of the two variables that according to the feminization literature are likely to differ the most between male and female physicians: number of hours worked in private sector and number of hours worked in public sector [10]. ZI models have been proposed to model count variables, dealing with an excess of zeros in several applications [26]. ZI models combine two components: one component is represented by a point mass at zero and another one is a count distribution, such as Poisson or negative binomial (the corresponding models are denoted by ZIP and ZINB, respectively). When the dependent variable tends to exhibit over-dispersion and/or an excess in number of zeros, ZI models present advantages with respect to classical multiple linear regression models. As public sector earnings are determined by class contracts and were not found to differ between men and women, multivariate analysis was not performed on this variable.

R Program (R Development Core Team 2008), through the function ZI of the pscl package [27], was used to fit this type of models. The MASS package was also used to fit the generalized linear models (GLMs) that typically are not sufficient for modelling excess zeros [28]. The Vuong test was used to verify if a GLM was indistinguishable from the corresponding ZI model [28, 29]. For hours worked in the public and in the private sector, we used GLMs because these variables did not present an excess number of zeros. For model fitting, the log-likelihood value and Akaike Information Criterion (AIC) were obtained. Pearson or deviance residuals [29] for different models were calculated to evaluate discrepancies between the observed and expected number of hours worked in private and/or public sectors predicted by each model. As the impact on workload of each individual year of practice was rather small, we assigned “years worked as a medical doctors” to 5-year increments to construct a continuous variable to facilitate its interpretation, which is an artefact often used in epidemiology [30].

## Results

### Proportion and characteristics of female physicians in the three cities

Across our 331 urban physicians, 46.2% were female, with the proportion being the highest in Praia (56.4%) and lowest in Bissau (28.1%). Overall, these were younger than their male peers in the three locations (*P* = 0.001), had more dependents (*P* = 0.034), were more likely to have a physician in the family (*P* < 0.001) but less likely to hold a specialty (*P* = 0.044) (Table 2).

**Table 2:** **Physician sample’s characteristics by city and gender**

	**Overall (331)**			**Praia (** ***n*** **= 110)**			**Maputo (** ***n*** **= 125)**			**Bissau (** ***n*** **= 96)**		
**Physician characteristics**	**M (** ***n*** **= 178)**	**F (** ***n*** **= 153)**	**P**	**M (** ***n*** **= 48)**	**F (** ***n*** **= 62)**	**P**	**M (** ***n*** **= 61)**	**F (** ***n*** **= 64)**	**P**	**M (** ***n*** **= 69)**	**F (** ***n*** **= 27)**	**P**
Age (median years)	47	37	0.001	46	36	0.001	45	37	<0.001	49	48	0.11
Dependents (median)	4	2	<0.001	2	1	0.032	3	2	0.049	8	4	<0.001
Married (% yes)	81.4%	64.1%	0.368	68.8%	59.7%	0.327	80.3%	73.4%	0.212	91.2%	51.9%	<0.001
Having a physician in the family	35.0%	60.3%	<0.001	51.1%	58.7%	0.158	29.5%	50.8%	0.016	29.0%	73.1%	<0.001
Working as a physician also outside the capital	18.6%	15.0%	0.657	27.7%	12.9%	0.053	19.7%	18.8%	0.896	11.6%	11.1%	1.000(^a^)
Holding a specialization	76.3%	52.3%	0.004	74.5%	58.1%	0.075	70.5%	42.2%	0.001	82.6%	63.0%	0.039
Public sector only	41.8%	42.8%	0.254	31.%	38.7%	0.259	36.1%	48.4%	0.373	53.6%	38.5%	0.008
Private sector only	10.2%	12.5%	0.657	17.0%	9.7%	0.373	4.9%	7.8%	0.260	10.1%	30.8%	0.165
Dual practice	48.0%	44.7%	0.456	51.1%	51.6%	0.345	50.0%	43.8%	0.567	36.2%	30.8%	0.458
Weekly working hours (public and private)	53.88	50.23	0.025	52.98	51.11	0.530	51.40	49.54	0.522	56.77	49.81	0.008
Public sector pay (2012 USD)	–	–	–	1472.48	1362.87	0.144	1056.15	887.36	0.030	386.96	360.12	0.577

Note: quantitative variables: Mann–Whitney test; qualitative variables: chi-square test or the alternative (^a^) Fisher exact test.

**Figure 1:**
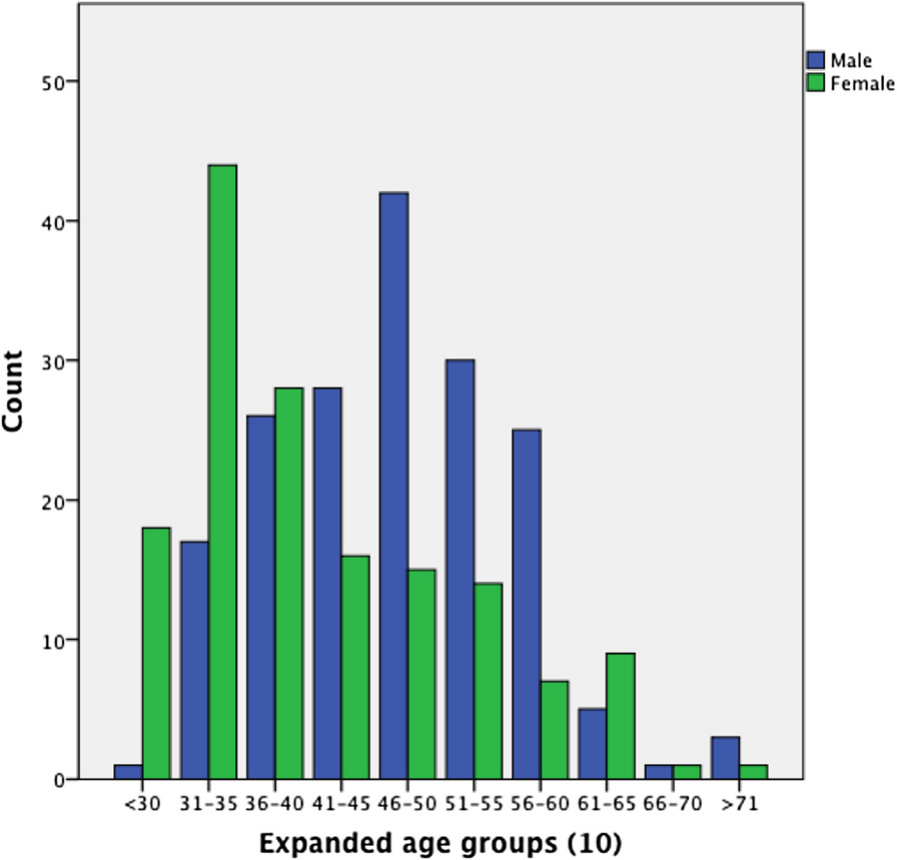
**Age distribution of physician population in the three locations, by gender.**

Median age for women in our sample was 37 years, as opposed to 47 years for men (*P* = 0.001). This difference of about 10 years in age was marked in Maputo and Praia; however, in Bissau, women and men presented similar median ages (*P* = 0.11). In each city, significant differences were found in the proportions of women in age groups, using the chi-square test (Praia: *P* = 0.009; Maputo: *P* < 0.001) and Fisher exact test for Bissau (*P* = 0.001). This pattern shows a dominance of female physicians in age group ≤35 years and of male ones in the older group (>50 years) in each city. When considering expanded age groups, in the three cities, female physicians were the majority (67.2%) in the three younger groups but only 28.4% of groups above 41 years of age (Fig. 1).

### Female physicians’ engagement with the profession

Overall, 52.3% of female physicians declared holding a specialty, as opposed to 76.3% of males, with the highest proportion recorded for Bissau (63%). Paediatrics, general practice and gynaecology were the most frequent specialties (8.5%, 6.5% and 5.2%, respectively); in total, female doctors represented 76.5% of paediatricians. By contrast, they were practically absent from surgery, orthopaedics, stomatology and otorhinolaryngology (Additional file 1: Table S3 in the statistical annex).

Working simultaneously in the public and private sectors was the preferred work modality for both male and female physicians in the three locations. The magnitude of dual practice was 44.7% for women (CI95% [37.1; 52.5]) and 48.0% for men (CI95% [40.8; 55.3]), using Wilson and Agresti–Coull methods. Forty-two per cent of female physicians reported working exclusively for the public sector, 12.5% exclusively for the private and the rest (44.7%) engaging in dual practice. No significant difference was found with sector engagement between genders.

Physician median net public salary per month was USD 1387 in Praia, USD 853 in Maputo and USD 335 in Bissau. Only in Maputo significant salary differences were found between men and women (*P* = 0.030), although such differences are likely due to men’s higher level of seniority. Among females working in the public, 30.3% declared receiving extra income from private services offered within public facilities, as opposed to 34.6% of their male peers. The situation was reversed in Bissau, where 50% of female and 39.6% of male physicians reported receiving extra income.

### Workloads

Female physicians worked shorter hours per week (50.23) than their male colleagues (53.88) (*P* = 0.025), with hours spent in the private sector accounting for the majority of the difference (9.71 vs. 12.57).

When considering the joint effect of all the relevant factors, Table 3 presents a GLM based on negative binomial. Being female and years of practice had both a significant and negative effect on the overall number of hours worked both in public and private (respectively, *P* = 0.024 and *P* = 0.0184). This indicates that female physicians present a decrease of about 7.8% in weekly working hours in comparison to their male peers and that each additional 5 years of practice also decreases the weekly hours worked by 2%. Having dependents has a positive effect on total hours worked (a 10.3% increase) but only at a 10% confidence level (*P* = 0.071). However, the adjustment of the GLM model to the data requires some cautions taking into account some trend in residuals (Table 3).

**Table 3:** **GLM on hours worked per week in public and in private sector**

	**Estimates**	**Std. error**	**z value**	**P value**
(Intercept)	3.998	0.070	57.057	<0.001**
City (Maputo, Mozambique)	−0.039	0.038	−1.013	0.3113
City (Bissau, Guinea-Bissau)	0.039	0.041	0.942	0.3460
Q2 gender (sex: female)	−0.077	0.034	−2.255	0.0242*
Q3 civil status (not married)	−0.004	0.037	−0.101	0.9292
Q4 dependents (yes)	0.103	0.057	1.803	0.0715
Q8 specialization (yes)	−0.062	0.038	−1.637	0.1016
Q6 years as a medical doctor (by 5-year increases)	−0.023	0.001	−2.358	0.0184*

*: *P* < 0.05; **: *P* < 0.001; *P* = 0.10 AIC = 2557.9.

Deviance residuals: min. −4.29, max. 2.83; median: −0.029.

When analysing separately the determinants of hours worked in public from those worked in private, the ZI models were preferred to GLM taking into account the amount of zeros. In the private sector, the ZINB model showed for the count component (that is, a number of hours >0) that being based in Bissau – with Praia as reference category (*P* < 0.001) – and not being married (*P* = 0.010) have a significant positive influence on physicians’ weekly private sector hours (Additional file 1: Table S4 in statistical annex). On the other hand, being female recorded a negative effect on hours worked in private (*P* = 0.001).

As for hours worked in the public sector, for the count component, the ZINB model shows a negative impact of having a specialty (*P* = 0.026) and years of practice (*P* = 0.003) – by 5-year unit – on hours worked in public, but no significant effect for sex (Additional file 1: Tables S5 in the statistical annex).

## Discussion

Our study shows that although female physicians do not represent yet the majority of the medical workforce in the three cities under study, feminization of the profession may be already under way, as shown by their over-representation in the younger age groups. Female doctors also distribute unevenly across medical specialties and are absent from traditionally male-dominated areas such as surgery, orthopaedics and stomatology. Our data also show that they engage as much as their male peers in private services, although all in all they end up dedicating fewer hours to the profession, particularly in the public sector.

As we studied physicians from only three cities, it is difficult to generalize these findings to rural areas that are known to be often shunned by female physicians [11]. As general practice is not a specialty in all the three countries, we suspect that some of those who declared holding such a specialty had, in fact, none; likewise, we believe that some of those without a specialization erroneously declared holding one because in their daily routine they informally engaged in some specialized practice. As we only conducted secondary analysis of quantitative data collected for another study, some female physicians’ specific features and motivations remain under-explored, and specifically designed qualitative interviews would be needed to understand better the contours of this phenomenon. As our study is cross-sectional and takes a snapshot of the situation in 2012, its ability to predict a trend for the feminization of the workforce is limited. However, this work provides a basis for an informed discussion on the extent of the feminization of the medical workforce in low-income settings, on its consequences and on how this compares to what is happening in the industrialized world.

If the current gender distribution was to persist, female doctors may at some stage become a majority in Bissau, Maputo and Praia. This is broadly consistent with the trend observed since the 1970s in countries like Canada [5], the United States [14], the United Kingdom [31] and more recently in Japan and France [3, 32]. It is still unclear why women are being more attracted today to the profession than in the past, although for African countries their increasing participation in the formal labour market, together with the improvement of their access to schooling, could be playing a part [33]. More deeply rooted gender inequalities, as well as a cultural perspective that tends to attribute to men breadwinning roles and consequently better access to education and professions [34], could help explain the time lag in the development of this phenomenon in the three African cities.

Our findings are also consistent with studies from high-income countries showing that female physicians work fewer hours than their male peers [13] but seem to contradict those suggesting that younger physicians work less than older ones [35]. Having children is often quoted as one of the factors associated with reduced working hours for female physicians [15]; however, in our cases, male doctors were found to have significantly more dependents, and number of dependents was associated with longer working hours per week. As the number of dependents increases, there may be pressure on male breadwinners to increase their working hours to meet the household’s augmented economic needs and on female members to reduce theirs to attend to child-rearing-related tasks [36].

We did not find significant evidence of in-country differentials between male and female doctors’ public sector salaries, and we could not reject the hypothesis that the small differences found were more attributable to lower levels of seniority between the two groups. This seems at odds with results from other studies in high-income countries on the significant sex-related pay gap existing in the profession [14, 37]. A possible interpretation is that public sector salary brackets in the three countries under analysis are very low and compressed and tightly related to seniority [38]; this would reduce the scope for salary discrimination between male and female physicians within the same seniority level.

The issue of feminization of the medical profession in countries where the density of physicians is low deserves more attention, particularly considering its possible impact on availability and accessibility of health professionals. Obviously, if more women enter the profession and they tend to work shorter hours, the physician workforce will soon require to either expand or become more efficient in order to provide the same level of services [1]. As countries progress towards the objective of universal health coverage, demand for health services is projected to increase and generate needs for more physicians and other providers of service [39]. A labour market relying on a medical workforce with different characteristics, preferences and professional inclinations may not be able to achieve an equitable and affordable distribution of health services, particularly in low-income settings [40].

Our findings should not be used to advocate for male quotas in the medical profession, which has been shown to create distortions and inefficiencies with long-lasting consequences [41]. On the contrary, policymakers should be encouraged to plan their future health workforce taking into account the trends behind the growth of the proportion of women in medical practice, as suggested by some scholars [15, 39, 42, 43]. This may entail recruiting more students, developing incentives for choosing understaffed specialties or to practice in underserved regions and designing measures to retain women in the health labour market. Family-friendly measures and more flexible working conditions, including part-time work, should be considered to that effect [44]. Better sex-disaggregate data would help inform the debate on how to carry out such a task as well as on gender discrimination in the health workforce [43]. The only certainty is that ignoring the specificities of the behaviour and needs of female physicians entails the risk of not making an efficient utilization of their potential contribution to the provision of health services [45].

## Conclusions

That female physicians are becoming the majority of the medical workforce in high-income countries is already documented, but whether this is happening in low- and middle-income countries and what the consequences of the phenomenon will be is another matter. Our secondary analysis of primary survey data explored the proportion and characteristics of female physicians in three African cities, with the objective of gaining an insight on extent and features of feminization of the medical workforce in low-income environments.

Our results showed that, although female physicians do not represent yet the majority of the medical workforce, feminization of the profession is happening and that female doctors distribute unevenly across medical specialties and engage as much as their male peers in private healthcare services, although they end up dedicating fewer hours to the profession, particularly in the public sector. More research is needed to understand how this phenomenon affects rural areas. Research will also need to include a broader range of countries to explore the amplitude of feminization of the medical profession and its consequences. Our work, however, shows the value of exploring differences between female and male physicians in order to anticipate the impact for national health workforces and health systems in low- and middle-income countries.

## Additional file

Additional file 1: Table S1. Physician workforce gender distribution, per selected countries. **Table S2.** Variables used in the analysis and description. **Table S3.** Physicians’ distribution across specialties per gender across the three locations. (DOCX 38 kb)
