# Supplementary material for: Feminization of the medical workforce in low-income settings; findings from surveys in three African capital cities
Source: Hum Resour Health. 2015 Jul 31;13:64. doi: 10.1186/s12960-015-0064-9 (PMC4521355; doi:10.1186/s12960-015-0064-9)
Supplement: Additional file 1: Table S1. — Physician workforce gender distribution, per selected countries. Table S2. Variables used in the analysis and description. Table S3. Physicians’ distribution across specialties per gender across the three locations. (DOCX 38 kb) [file 12960_2015_64_MOESM1_ESM.docx]

# Statistical annex

Table S1: Physician workforce gender distribution, per selected countries

| Country | Year | Male | Female | Proportion of female |
| --- | --- | --- | --- | --- |
| Canada | 2003 | 44,514 | 22,069 | 33% |
| Cape Verde | 2004 | 112 | 119 | 52% |
| France | 2004 | 126,772 | 76,715 | 38% |
| Guinea Bissau | 2004 | 129 | 59 | 31% |
| Japan | 2002 | 212,744 | 39,145 | 16% |
| Mozambique | 2004 | 267 | 247 | 48% |
| United Kingdom | 1997 | 87,262 | 46,379 | 35% |

Source: Global health Workforce Statistics database, WHO Geneva

Table S2: Variables used in the analysis and description

| **Variable** | **Specific questionnaire question** |
| --- | --- |
| Age | Q1_Age |
| Female | Q2_Sex dummy: 1 is female and 0 is male |
| Q3_Unmarried | Q3_Civil status dummy: 1 is unmarried and 0 is married |
| No. dependents | Q4_Number of dependents |
| Years as physician^(a)^ | Q6_How long have you worked as medical doctor (years)? |
| Work outside the city | Q7_Do you currently work as physician outside the city you live? |
| Specialist | Q8_Do you hold any specialization in medicine? 1: No; 1: Yes, which one |
| Level of working institution in the public sector | Q12_What level of care do you currently work for? (multiple options) |
| Extra income from the public sector work | Q15_Do you get any other salary from your public sector work? 0: No; 1: Yes |
| Type of working institution in the private sector | Q19_What sort of private sector institution do you work for? (multiple options) |
| Weekly working hours private | Weekly working hours in the private sector (as from Tables B and F) |
| Weekly hours spent in the public sector | Weekly working hours in the private sector (as from Tables B and F) |
| Log wage public | Log monthly wage of the public sector (as from Tables B and F) |
| Log wage private | Log hourly wage of the private sector (as from Tables B and F) |

1. To facilitate the interpretation in GLM and zero inflated models, we also convert this variable to a 5-years unit.

Table S3: Physicians’ distribution across specialties per gender across the three locations

| Type of specialization | | Gender declared by physicians | | Total |
| --- | --- | --- | --- | --- |
|  |  | Male | Female |  |
| No specialisation | Count | 43 | 73 | 116 |
|  | % within Type of specialization | 37.10% | 62.90% | 100.00% |
| Anaesthesiology | Count | 4 | 6 | 10 |
|  | % within Type of specialization | 40.00% | 60.00% | 100.00% |
| Biochemistry | Count | 1 | 0 | 1 |
|  | % within Type of specialization | 100.00% | 0.00% | 100.00% |
| Cardiology | Count | 6 | 3 | 9 |
|  | % within Type of specialization | 66.70% | 33.30% | 100.00% |
| Surgery | Count | 21 | 1 | 22 |
|  | % within Type of specialization | 95.50% | 4.50% | 100.00% |
| Dermatology | Count | 0 | 4 | 4 |
|  | % within Type of specialization | 0.00% | 100.00% | 100.00% |
| Endocrinology | Count | 0 | 1 | 1 |
|  | % within Type of specialization | 0.00% | 100.00% | 100.00% |
| Entomology | Count | 1 | 0 | 1 |
|  | % within Type of specialization | 100.00% | 0.00% | 100.00% |
| Epidemiology | Count | 1 | 1 | 2 |
|  | % within Type of specialization | 50.00% | 50.00% | 100.00% |
| Stomatology | Count | 7 | 2 | 9 |
|  | % within Type of specialization | 77.80% | 22.20% | 100.00% |
| Pharmacology | Count | 0 | 1 | 1 |
|  | % within Type of specialization | 0.00% | 100.00% | 100.00% |
| Physiotherapy | Count | 0 | 1 | 1 |
|  | % within Type of specialization | 0.00% | 100.00% | 100.00% |
| Gastroenterology | Count | 2 | 0 | 2 |
|  | % within Type of specialization | 100.00% | 0.00% | 100.00% |
| Genecology | Count | 8 | 8 | 16 |
|  | % within Type of specialization | 50.00% | 50.00% | 100.00% |
| Haematology | Count | 1 | 0 | 1 |
|  | % within Type of specialization | 100.00% | 0.00% | 100.00% |
| Medical radiology | Count | 3 | 3 | 6 |
|  | % within Type of specialization | 50.00% | 50.00% | 100.00% |
| Immunology | Count | 1 | 2 | 3 |
|  | % within Type of specialization | 33.30% | 66.70% | 100.00% |
| Infectology | Count | 3 | 1 | 4 |
|  | % within Type of specialization | 100.00% | 0.00% | 100.00% |
| General practice | Count | 7 | 10 | 17 |
|  | % within Type of specialization | 41.20% | 58.80% | 100.00% |
| Internal medicine | Count | 10 | 4 | 14 |
|  | % within Type of specialization | 71.40% | 28.60% | 100.00% |
| Anatomo pathology | Count | 5 | 4 | 9 |
|  | % within Type of specialization | 55.60% | 44.40% | 100.00% |
| Nephrology | Count | 1 | 0 | 1 |
|  | % within Type of specialization | 100.00% | 0.00% | 100.00% |
| Neurology | Count | 3 | 1 | 4 |
|  | % within Type of specialization | 75.00% | 25.00% | 100.00% |
| Ophthalmology | Count | 5 | 3 | 8 |
|  | % within Type of specialization | 62.50% | 37.50% | 100.00% |
| Oncology | Count | 0 | 1 | 1 |
|  | % within Type of specialization | 0.00% | 100.00% | 100.00% |
| Orthopaedics | Count | 10 | 0 | 10 |
|  | % within Type of specialization | 100.00% | 0.00% | 100.00% |
| Otorhinolaryngology | Count | 5 | 1 | 6 |
|  | % within Type of specialization | 83.30% | 16.70% | 100.00% |
| Paediatrics | Count | 4 | 13 | 17 |
|  | % within Type of specialization | 23.50% | 76.50% | 100.00% |
| Anemology | Count | 1 | 2 | 3 |
|  | % within Type of specialization | 33.30% | 66.70% | 100.00% |
| Psychiatry | Count | 4 | 1 | 5 |
|  | % within Type of specialization | 80.00% | 20.00% | 100.00% |
| Public Health | Count | 19 | 5 | 24 |
|  | % within Type of specialization | 79.20% | 20.80% | 100.00% |
| Urology | Count | 2 | 1 | 3 |
|  | % within Type of specialization | 66.70% | 33.30% | 100.00% |
| Total | Count | 178 | 153 | 331 |
|  | % within Type of specialization | 53.80% | 46.20% | 100.00% |

Table S4: ZINB model for hours worked in the private sector

|  |  | Count model coefficients | | | Zero-inflation model coefficients | | | |
| --- | --- | --- | --- | --- | --- | --- | --- | --- |
|  | Est | SE | Z-value | p | Est | SE | Z-value | *p* |
| Intercept | 2.97783 | 0.27749 | 0.27749 | <2e-16 *** | 0.464196 | 0.550896 | 0.843 | 0.39944 |
| cityMaputo (Mozambique | -0.15803 | 0.14451 | -1.094 | 0.274138 | 0.286697 | 0.299747 | 0.956 | 0.33884 |
| cityBissau (Guinea Bissau) | 0.53178 | 0.14910 | 3.567 | 0.000362 *** | 0.796384 | 0.319785 | 2.490 | 0.01276 * |
| q2_sexFemale | -0.39060 | 0.12168 | -3.210 | 0.001326 ** | -0.083886 | 0.264649 | -0.317 | 0.75127 |
| q3_civil_statusNot married | 0.34592 | 0.13497 | 2.563 | 0.010377 * | -0.008541 | 0.290877 | -0.029 | 0.97657 |
| q4_dependentsYes | 0.08626 | 0.21166 | 0.408 | 0.683617 | -0.149057 | 0.457551 | -0.326 | 0.74460 |
| q8_specializationYes | -0.08112 | 0.15484 | -0.524 | 0.600345 | -0.930750 | 0.288191 | -3.230 | 0.00124 ** |
| q6_years as a medical doctor (by 5-year increases) | -0.03073 | 0.03871 | -0.794 | 0.427311 | -0.138727 | 138727 0.081321 | 1.706 | 0.08802 . |

---

Signif. codes: 0 '***' 0.001 '**' 0.01 '*' 0.05 '.' 0.1 ' ' 1

Table S5: ZINB model for hours worked in the public sector

|  |  | Count model coefficients | | | Zero-inflation model coefficients | | | |
| --- | --- | --- | --- | --- | --- | --- | --- | --- |
|  | Est | SE | Z-value | *p* | Est | SE | *Z-value* | *p* |
| Intercept | 4.07676 | 0.08117 | 50.222 | < 2e-16 *** | -1.58017 | 0.71242 | -2.218 | 0.0266 * |
| cityMaputo (Mozambique) | -0.05117 | 0.04229 | -1.210 | 0.22626 | -0.95685 | 0.55448 | -1.726 | 0.0844 . |
| cityBissau (Guinea Bissau) | 0.06513 | 0.04891 | 1.332 | 0.18299 | 0.49498 | 0.43341 | 1.142 | 0.2534 |
| q2_sexFemale | -0.01872 | 0.03860 | -0.485 | 0.62765 | -0.04762 | 0.41569 | -0.115 | 0.9088 |
| q3_civil_statusNot married | -0.03574 | 0.04251 | -0.841 | 0.40054 | 0.50691 | 0.43107 | 1.176 | 0.2396 |
| q4_dependentsYes | -0.09405 | 0.06893 | 1.364 | 0.17243 | -1.25894 | 0.52598 | -2.393 | 0.0167 * |
| q8_specializationYes | -0.09482 | 0.04264 | -2.224 | 0.02615 * | -1.25894 | 0.52598 | -2.393 | 0.2495 |
| q6_years as a medical doctor (by 5-year increases) | -0.03253 | 0.01110 | -2.932 | 0.00337 ** | 0.56888 | 0.49398 | 1.152 | 0.2495 |

---

Signif. codes: 0 '***' 0.001 '**' 0.01 '*' 0.05 '.' 0.1 ' ' 1
